# Analysis of Failure to Achieve the Critical View of Safety (CVS) in Laparoscopic Cholecystectomy: A Prospective Observational Study

**DOI:** 10.7759/cureus.111481

**Published:** 2026-06-25

**Authors:** S. Sriram Kumar, Vishal Gupta, Ajeet P Maurya, Radha S Gupta

**Affiliations:** 1 Department of Surgical Gastroenterology, All India Institute of Medical Sciences, Bhopal, IND; 2 Department of General Surgery, All India Institute of Medical Sciences, Bhopal, IND; 3 Department of Radiodiagnosis, All India Institute of Medical Sciences, Bhopal, IND

**Keywords:** biliary injury, conventional laparoscopic cholecystectomy, critical view of safety (cvs), gallbladder stones, laparoscopic gallbladder surgery, post-cholecystectomy bile duct injury

## Abstract

Background

The critical view of safety (CVS) is the principal intraoperative strategy to prevent bile duct injury during laparoscopic cholecystectomy (LC). However, CVS may not be achieved in all cases. Factors that predict such failure remain understudied. The aim of this study was to determine the rate of failure to achieve CVS during LC and to identify various preoperative and intraoperative predictors of failure to achieve CVS.

Methods

In this prospective observational study, 41 adult patients undergoing LC for gallstone disease at a single center were included. LC was performed using the standard four-port technique by a single hepatobiliary surgeon utilizing the CVS approach. Various preoperative variables, including the Gall Bladder Reporting and Data System (GB-RADS) score and Cholecystectomy Laparoscopic-to-Open Conversion (CLOC) score, and intraoperative variables, including the modified Nassar grade, were recorded. Univariable tests and multivariable logistic regression analysis were performed to determine various predictors of failure to achieve CVS.

Results

A total of 41 patients with a mean age of 41.66±11.74 years were included in the study. There were 30 (73.2%) females. The most common indication for LC was biliary colic in 25 (60.9%) cases. The CVS could not be achieved in 6 (14.63%) cases. These six failures were managed with subtotal cholecystectomy (laparoscopically in four and open in two). The overall conversion rate was 2 (4.87%). On univariable analysis, raised serum alkaline phosphatase (p=0.012) and serum gamma-glutamyl transferase (p=0.047) as preoperative laboratory parameters, the presence of pericholecystic fluid (p=0.026) and a higher GB-RADS score (p=0.010) as imaging features, and limited extension of the gallbladder fundus in relation to the liver edge (till/short of liver edge) (p=0.008) and a fibrotic gallbladder bed (p < 0.005) as intraoperative findings were factors found to be associated with failure to achieve CVS. On multivariable analysis, a GB-RADS score of 2 (odds ratio (OR) 8.74) and a fibrotic gallbladder bed (OR 10.04) were found to be independent predictors of failure to achieve CVS. The total CLOC score was independently predictive (OR 26.6) and discriminated failure to achieve CVS better than the modified Nassar grade (area under the curve, 0.788 vs. 0.704).

Conclusions

The CVS can be achieved in a majority of cases during LC. A higher GB-RADS score, a fibrotic gallbladder bed, and a higher CLOC score can identify at-risk patients and may aid preoperative planning and readiness for early bailout.

## Introduction

Laparoscopic cholecystectomy (LC) may carry a higher risk of bile duct injury (BDI) than open cholecystectomy despite being the standard-of-care procedure for gallstones [1]. Bile duct injury is a serious, morbid, and costly complication of LC [1]. A major BDI may be associated with substantial long-term consequences, including the need for multiple interventions and complex biliary reconstruction, recurrent cholangitis, the need for reintervention if the biliary repair fails, diminished long-term survival, and compromised quality of life, besides the associated medicolegal risk for the surgeon [1-3].

Understanding the factors involved in BDI during LC suggests that most biliary injuries result from misidentification of the key structures; the common bile duct (CBD) is misidentified as the cystic duct and divided [4]. To prevent such misidentification injuries, Strasberg et al. [4] introduced the concept of the critical view of safety (CVS) as a method for conclusive identification of the cystic duct and artery before their division [4,5]. The CVS is now the central element of the “Culture of Safety in Cholecystectomy” and is endorsed by almost all international societies and their guidelines [6-9].

Although the CVS can be attained in the majority of cases, it may not be possible to achieve it in a small proportion of cases (around 10%) [10,11]. Failure to achieve the CVS after a reasonable attempt signals a genuinely difficult and potentially dangerous situation in which continued dissection in the hepatocystic triangle risks bile duct or vascular injury, thus necessitating resorting to a bailout method for safe completion of the procedure [12].

Preoperative identification of predictors of failure to achieve CVS may help improve case selection, patient counseling, and operative planning. However, data specifically addressing this aspect of the CVS remain limited [11,13,14].

This study aimed to prospectively analyze the rate of failure to achieve the CVS during LC and the various factors that may predict such failure.

## Materials and methods

Study design

This prospective observational study was conducted from August 2024 to February 2026. The study was approved by the Institutional Ethics Committee (Ref. No. ihecsr/aiimsbhopal/aug/65). Written informed consent was obtained from every participant before enrollment. The study is reported in accordance with the Strengthening the Reporting of Observational Studies in Epidemiology (STROBE) statement for observational studies [15].

Participants

Consecutive adult patients (≥18 years) with gallstone disease requiring LC and fit for general anesthesia were included in the study. Patients with suspected gallbladder carcinoma, polyp, or adenomyomatosis without stones, Mirizzi syndrome, advanced cirrhosis (Child-Pugh-Turcotte class B or C), and those planned for upfront open cholecystectomy or cholecystectomy concomitant with another abdominal procedure, unfit for general anesthesia, or who declined consent were excluded.

Sample size

The sample size was calculated using the formula n=4pq/d²; the p value was the expected CVS non-achievement rate, taken as 10% as reported in the literature [10], and the q value was 1-p. With an absolute precision (d) of 10%, the calculated sample size (n) needed for the study was 36.

Pre-operative assessment

Demographic, clinical, laboratory, radiological, operative, and perioperative data were recorded prospectively on a structured proforma. Clinical variables included the duration of symptoms, previous interventions, and the history of prior acute cholecystitis, acute pancreatitis, or endoscopic retrograde cholangiopancreatography (ERCP). Patients with acute cholecystitis underwent elective interval cholecystectomy after recovery following initial conservative management at the time of acute presentation.

Laboratory evaluation comprised a complete blood count, liver and renal function tests, and C-reactive protein. All patients underwent abdominal ultrasonography within one week of surgery, assessing various relevant parameters, including stone number and size, the presence of a stone impacted in the neck, gallbladder appearance (distended or contracted), gallbladder wall thickness, and pericholecystic changes. The Gall Bladder Reporting and Data System (GB-RADS) score was also assessed [16]. Magnetic resonance cholangiopancreatography and/or computed tomography were obtained when indicated (e.g., acute cholecystitis, a thick-walled gallbladder, or suspected CBD stones). The preoperative Cholecystectomy Laparoscopic-to-Open Conversion (CLOC) score was calculated within one week of surgery based on various parameters, e.g., age, sex, indication, American Society of Anesthesiologists (ASA) class, and ultrasonographic gallbladder wall and CBD findings [17].

Surgical procedure and assessment of the intraoperative variables

All operations were performed under general anesthesia using a standard four-port American technique by a single surgeon. The CVS, with its anterior and posterior (doublet) views, was used to identify the cystic duct and cystic artery [4,18]. When the CVS was found difficult to achieve on the initial attempt, a reattempt was made after adjunct maneuvers (gallbladder aspiration, dislodging an impacted neck stone, proper exposure, and taking a deliberate time-out). Conventional intraoperative cholangiography was not used. Indocyanine green (ICG) near-infrared fluorescence cholangiography was used when available (ICG dose: 0.05 mg/kg, intravenously one to three hours preoperatively). The procedure was video-recorded for assessment of the CVS and other intraoperative findings. The CVS was scored in real time by the operating surgeon using the six-point Strasberg scale and was considered satisfactory at a score of at least 5 out of 6 [18]. The CVS was declared achieved based on this score, and after a time-out session and discussion with another surgeon (a senior fellow) of the operating team. The CVS that could not be achieved after a reasonable attempt, i.e., even after another attempt using the adjunct maneuvers mentioned above, was declared as CVS not achieved.

Intraoperative variables assessed included the modified Nassar operative-difficulty grade [11], extension of the gallbladder fundus in relation to the liver edge (i.e., the fundus extends beyond the liver edge or remains confined to or short of the liver edge on retraction), nature of the gallbladder bed (normal vs. fibrotic, i.e., the cystic plate fused to the gallbladder due to inflammation) [8], the pucker sign (i.e., indentation or dimpling on the edge of the liver adjacent to the fundus) [19], pericholecystic adhesions, pericholecystic edema, condition of Calot’s triangle, presence of impacted stone in the neck, mucocele or empyema, and identification of various relevant anatomical landmarks.

Similar to the CVS, various important intraoperative findings, such as a fibrotic gallbladder bed, liver pucker sign, and gallbladder fundus extension relative to the liver edge, were assessed after a time-out session and discussion with another surgeon (a senior fellow) of the operating team.

Outcomes

The primary outcome was the rate of failure to achieve the CVS after a reasonable attempt. Perioperative outcomes analyzed were operative time, blood loss, open conversion rate, BDI rate, 30-day complications (overall and as graded by the Clavien-Dindo classification), hospital stay, and mortality.

Statistical analysis

Data were analyzed with IBM SPSS Statistics v26 (IBM Corp., Armonk, NY, USA). Quantitative variables were summarized as mean, median, and range, and categorical variables as numbers and proportions. Patients in whom the CVS was achieved (CVS-Yes group) were compared with those in whom it could not be achieved (CVS-No group) using the chi-square or Fisher’s exact test for categorical variables and the Student’s t-test for continuous variables with normal distribution and the Mann-Whitney U test for continuous variables with non-normal distribution. A two-sided p<0.05 was considered significant. Variables significant on univariable analysis were entered into a multivariable binary logistic regression model to identify independent predictors of failure to achieve CVS, reported as adjusted odds ratios (OR) with 95% confidence intervals (CI). A separate score-based model evaluated the total CLOC score and the modified Nassar grade. To avoid overfitting, given the limited number of outcome events, the number of variables entered into each model was deliberately restricted. Model performance was assessed with the omnibus test of model coefficients, the Nagelkerke R², and the Hosmer-Lemeshow goodness-of-fit test. The discriminative ability of the total CLOC score and the modified Nassar grade was assessed by receiver operating characteristic analysis, with the area under the curve and optimal cutoffs reported.

## Results

Demographic and clinical characteristics

Forty-one patients with a mean age of 41.66 years were included in the present study. The majority, 30 (73.2%), were female, and 32 (78%) were aged ≤50 years. The mean body mass index (BMI) of the entire cohort was 24.21 kg/m², with a range of 16.2-34.2 kg/m². A history of prior abdominal surgery was present in 23 (56.1%) patients, including an abandoned LC in one. Biliary colic was the most common indication for LC, present in 25 (60.9%) patients. None of the patients with acute cholecystitis were operated on during the acute phase; rather, they underwent elective interval cholecystectomy after being managed conservatively during the acute presentation. Baseline laboratory parameters (complete blood counts and liver function tests) were largely normal except for slightly elevated C-reactive protein (mean±SD, 8.23±32.44 mg/L; normal range <5 mg/L).

On ultrasonography, multiple gallstones were present in 27 (65.9%) patients, and all patients had a GB-RADS score of 1 or 2. A preoperative CLOC score of 6 or less (low conversion risk) was present in 37 (90.2%). Further details are shown in Table 1.

**Table 1:** Baseline demographic, clinical, and imaging characteristics (N=41) The data are presented as n (%) or mean±SD. No formal statistical comparisons were performed for baseline variables. *The mixed group included four patients who had more than one indication for surgery. Two patients had acute cholecystitis after the resolution of acute biliary pancreatitis. Two patients continued to have biliary colic after endoscopic clearance of CBD stones. CLOC, Cholecystectomy Laparoscopic-to-Open Conversion; ASA, American Society of Anesthesiologists; CBD, common bile duct; GB-RADS, Gall Bladder Reporting and Data System; GB, gallbladder

S. no.	Parameter	Category	Result
	Clinical characteristics		
1	Age (years)		41.66±11.74
2	Age ≤50 years		32 (78)
3	Sex	Female	30 (73.2)
		Male	11 (26.8)
4	Previous abdominal surgery		23 (56.1)
5	Body mass index (kg/m^2^)	Overall	24.21±3.36
		>25 kg/m²	27 (65.9)
6	ASA class I		41 (100)
7	Primary indication for cholecystectomy	Biliary colic	25 (60.9)
		Resolved acute cholecystitis	7 (17)
		Acute biliary pancreatitis	1 (2.4)
		CBD stones	2 (4.8)
		Mixed*	4 (9.7)
		Asymptomatic (Incidental)	2 (4.9)
8	Duration of symptoms (months)		15.24±12.73
9	CLOC score	≤6	37 (90.2)
		>6	4 (9.7)
	Imaging characteristics		
10	Stone numbers	Single	12 (29.3)
		Multiple	27 (65.9)
		Sludge	2 (4.9)
11	Largest stone size	<2 cm	36 (92.3)
		>2 cm	3 (7.7)
12	Impacted stone in neck		1 (2.4)
13	GB wall thickness	<3 mm	34 (82.9)
		>3 mm	7 (17.1)
14	Pericholecystic fluid	Yes	2 (4.9)
		No	39 (95.1)
15	GB-RADS score	1	34 (82.9)
		2	7 (17.1)

Operative findings

Anatomical landmarks were identified in most cases, including Rouvière’s sulcus in 32 (78%). Most operations were of low difficulty, with modified Nassar grades 1-2 noted in 32 (78%) patients. Fibrotic pericholecystic adhesions were present in 15 (36.6%), an abnormal Calot’s triangle in 11 (26.8%), and a fibrotic gallbladder bed in 10 (24.4%) patients. Further details are shown in Table 2.

**Table 2:** Intraoperative findings during LC (N=41) The data are presented as n (%). No formal statistical comparisons were performed for baseline variables. LC, laparoscopic cholecystectomy

S. no.	Parameter	Category	Result, n (%)
1	Modified Nassar grade	1	19 (46.3)
		2	13 (31.7)
		3	3 (7.3)
		4	6 (14.6)
2	Gallbladder extension (fundus)	Beyond the liver edge	19 (46.3)
		Till the liver edge	18 (43.9)
		Short of the liver edge	4 (9.8)
3	Impacted stone in neck/Hartmann’s pouch	-	6 (14.6)
4	Mucocele gallbladder	-	3 (7.3)
5	Empyema gallbladder	-	2 (4.9)
6	Peri-cholecystic fibrotic adhesions	-	15 (36.6)
7	Peri-cholecystic edema	-	10 (24.4)
8	Calot’s triangle	Normal	30 (73.2)
		Fibrotic	11 (26.8)
9	Gallbladder bed	Normal	31 (75.6)
		Fibrotic	10 (24.4)
10	Liver status	Normal	37 (90.2)
		Fatty	4 (9.8)
11	Identification of various anatomical landmarks	Common bile duct	40 (97.6)
		Rouvière’s sulcus	32 (78)
		Hepatic artery proper	39 (95.1)
		Umbilical fissure	40 (97.6)

Perioperative outcomes

**Figure 1:**
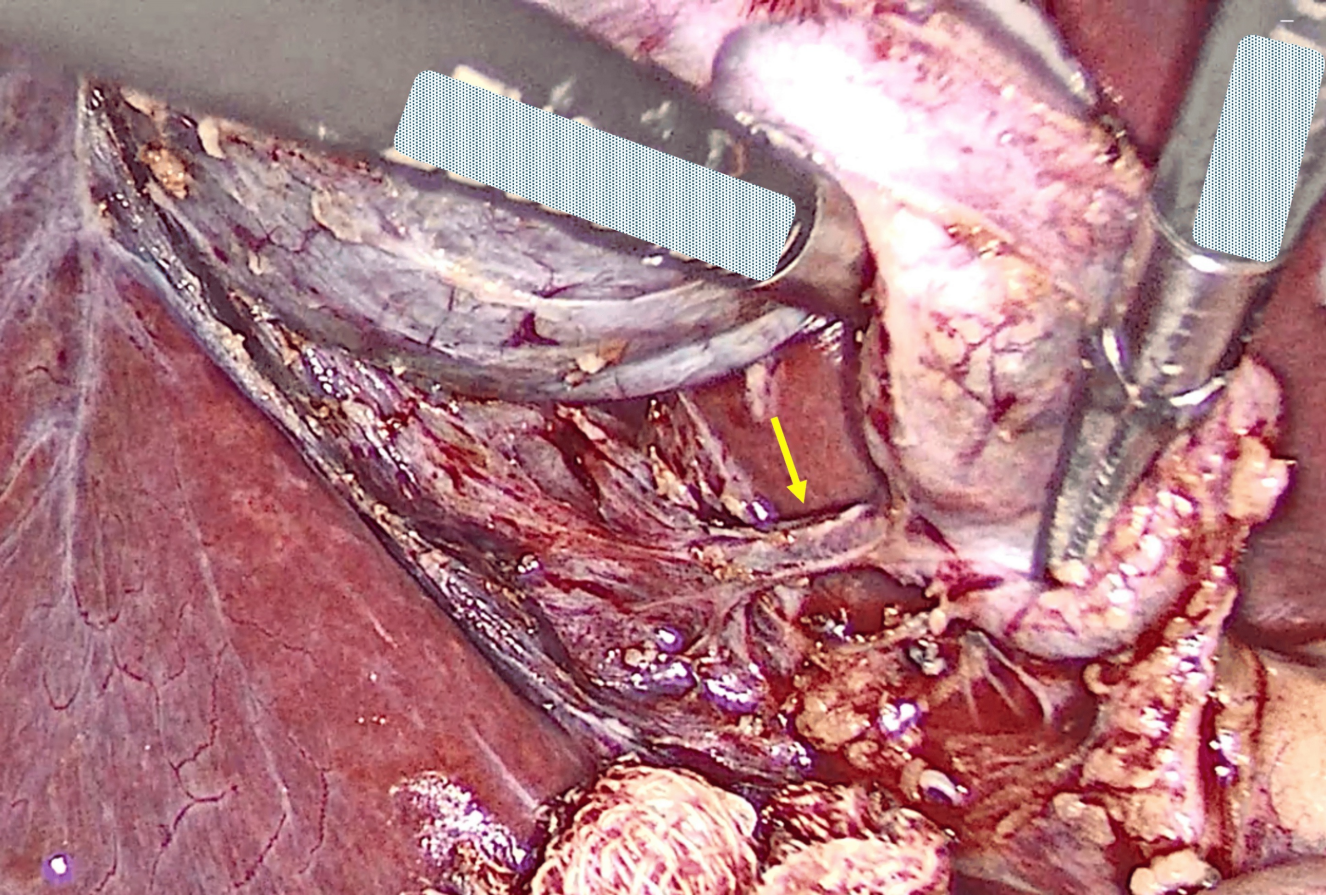
Aberrant bile duct (yellow arrow) draining into the gallbladder near the neck; reconstituting STC was performed to preserve the aberrant duct STC, subtotal cholecystectomy

The CVS was attempted in all 41 patients and was achieved in 35 (85.36%), resulting in a failure rate of 14.63%. The reason for failure to obtain the CVS was dense fibrosis in Calot’s triangle in all cases. Two of these cases had prior acute cholecystitis, and three had prior biliary intervention (ERCP) for CBD stones. A bailout procedure was required in seven (17%) patients, including open conversion in two patients. The final bailout procedure was subtotal cholecystectomy (STC). All STCs were of the reconstituting subtype. Six of these seven bailouts were performed because of failure to achieve the CVS. However, in the remaining patient, STC was performed despite achieving the CVS because of aberrant bile duct anatomy (Figure 1). Indocyanine green fluorescence cholangiography was used in four patients, all of whom attained the CVS.

Two patients (both after STC) had minor bile leaks postoperatively that resolved with conservative management without any further intervention. On histopathology, 39 (95.12%) patients were diagnosed with chronic cholecystitis, and two with acute-on-chronic cholecystitis. One of the patients with chronic cholecystitis had associated moderate dysplasia. Further details on perioperative outcomes are shown in Table 3.

**Table 3:** Perioperative outcomes of entire cohort (N=41) The data are presented as n (%), mean±SD, or median (interquartile range). No formal statistical comparisons were performed for baseline variables. *All STCs were of the reconstituting subtype. CVS, critical view of safety; CD, Clavien-Dindo morbidity grade; BDI, bile duct injury; STC, subtotal cholecystectomy

S. no.	Outcome variables		Result
1	CVS achieved		35 (85.4)
2	CVS not achieved		6 (14.6)
3	Bailout procedure	Total	7 (17)
		Laparoscopic STC*	5 (12.2)
		Open STC*	2 (4.9)
4	Open conversion		2 (4.9)
5	BDI		0 (0)
6	Minor bile leak		2 (4.9)
7	Blood loss (mL)		20 (10-50)
8	Duration of surgery (minutes)		153.17±46.93
9	Postoperative hospital stays (days)		3 (2-4)
10	30-day complications (overall)		2 (4.87)
11	30-day complications (CD grade >2)		0 (0)
12	30-day mortality		0 (0)

Overall postoperative complications were more frequent in the failure-to-obtain-CVS (CVS-No) group (p=0.024), with higher operative time (p<0.001), blood loss (p=0.038), and postoperative stay (p=0.004) (Table 4).

**Table 4:** Perioperative outcomes in relation to failure to achieve CVS The data are presented as n (%), mean±SD, or median (interquartile range). ^#^Non-normally distributed continuous outcomes were analyzed using the Mann-Whitney U test. ^†^Normally distributed continuous outcomes were analyzed using independent t-tests. ^††^Overall 30-day complications were compared using Fisher’s exact test. *Statistically significant at p<0.05. CD: Clavien-Dindo morbidity grade; CVS, critical view of safety

S. no.	Parameter	CVS-Yes (N=35)	CVS-No (N=6)	Test value	P-value
1	Blood loss (mL)^#^	20 (10-50)	50 (50-50)	158.5	p=0.038*
2	Duration of surgery (minutes)^†^	142.1±41.3	210.6±25.4	-3.91	p<0.001*
3	Postoperative hospital stays (days)^#^	3 (2-4)	5.5 (4-8)	180.5	p=0.004*
4	Overall 30-day complications^††^	0 (0)	2 (33.33)	-	p=0.024*
5	30-day complications CD grade >2	0 (0)	0 (0)	-	-
6	30-day mortality	0 (0)	0 (0)	-	-

Predictors of failure to achieve the critical view of safety

Univariable Analysis

On univariable analysis, a CLOC score greater than six (p=0.055) and the presence of a CBD stent (p=0.070) were found to be borderline associated with failure to achieve CVS. Various demographic and clinical variables were not found to be significantly associated with the inability to achieve CVS, except for duration of symptoms, which was shorter in the CVS-No group. Further details are shown in Table 5.

**Table 5:** Predictors of failure to achieve CVS: univariable analysis of demographic and clinical parameters The data are presented as n (%) or mean±SD. ^†^Age, BMI, and duration of symptoms were compared using independent t-tests, while the remaining variables were analyzed using Fisher’s exact test. ^#^Statistically significant at p<0.05. BMI, body mass index; ERCP, endoscopic retrograde cholangiopancreatography; CBD, common bile duct; CVS, critical view of safety; CLOC, Cholecystectomy Laparoscopic-to-Open Conversion

S. no.	Variable	Category	Total (N=41)	CVS-Yes (N=35)	CVS-No (N=6)	Test value	P-value
1	Age	-	-	41.96±11.83	46.38±12.47	-0.85^†^	0.138
2	BMI	-	-	24.22±3.47	24.26±3.03	-0.03^†^	0.854
3	Sex	Male	11	9 (25.71)	2 (33.33)	-	1.000
		Female	30	26 (74.29)	4 (66.67)		
4	Duration of symptoms			16.37±13.34	8.83±5.53	2.35^†^	0.03^#^
5	Biliary colic	Yes	30	26 (74.29)	4 (66.67)	-	1.000
		No	11	9 (25.71)	2 (33.33)		
6	Prior acute cholecystitis	Yes	10	7 (20)	3 (50)	-	0.331
		No	31	28 (80)	3 (50)		
7	Prior acute biliary pancreatitis	Yes	3	3 (8.57)	0 (0)	-	1.000
		No	38	32 (91.43)	6 (100)		
8	Prior ERCP (for CBD stones)	Yes	3	1 (2.86)	2 (33.33)	-	0.068
		No	38	34 (97.14)	4 (66.67)		
9	Previous surgery	Yes	23	20 (57.14)	3 (50)	-	1.000
		No	18	15 (42.86)	3 (50)		
10	Previous surgery site	Upper abdomen	4	3 (8.57)	1 (16.67)	-	0.999
		Lower abdomen	19	17 (48.57)	2 (33.33)		
11	CLOC score	>6	4	1 (2.86)	3 (50)	-	0.055
		≤6	37	34 (97.14)	3 (50)		

On analysis of various laboratory and imaging parameters, preoperative factors found to be significantly associated with failure to achieve CVS were higher serum alkaline phosphatase (SAP) (p=0.012) and gamma-glutamyl transferase (GGT) (p=0.047) levels, the presence of pericholecystic fluid (p=0.026) on imaging, and a GB-RADS score of two (p=0.010). Further details are shown in Table 6 and Table 7.

**Table 6:** Predictors of failure to achieve CVS: univariable analysis of laboratory parameters The data are presented as mean±SD or median (interquartile range). All variables were normally distributed except CRP. ^†^CRP was analyzed using the Mann-Whitney U test. The remaining variables were analyzed using independent t-tests. ^#^Statistically significant at p<0.05. TLC, total leukocyte count; CRP, C-reactive protein; SGOT, serum glutamic oxaloacetic transaminase; SGPT, serum glutamic pyruvic transaminase; ALP, alkaline phosphatase; GGT, gamma-glutamyl transferase; CVS, critical view of safety

S. no.	Variable	Unit	Reference range	CVS-Yes (N=35)	CVS-No (N=6)	Test value	P-value
1	Hemoglobin	g/dL	11-15	12.21±1.34	12.47±1.60	-0.43	0.578
2	TLC	10^3^/μL	4.00-11.00	8126±4809	7707±1853	0.42	0.809
3	CRP^†^	mg/L	<5	1.40 (0.90-1.92)	2.18 (1.75-3.19)	142	0.098
4	Total bilirubin	mg/dL	0.3-1.2	0.59±0.43	0.60±0.26	-0.08	0.314
5	SGOT	U/L	10-50	25.87±7.02	24±7.32	0.60	0.699
6	SGPT	U/L	10-50	23.59±12.61	29.54±10.98	-1.08	0.165
7	ALP	U/L	40-129	75.38±21.58	109.73±40.07	-2.03	0.012^#^
8	GGT	U/L	<60	24.30±19.55	40.86±32.58	-1.23	0.047^#^
9	Albumin	g/dL	3.5-5.2	4.24±0.31	4.18±0.39	0.35	1.000

**Table 7:** Predictors of failure to achieve CVS: univariable analysis of imaging parameters The data are presented as n (%) or mean±SD. ^†^All variables were analyzed using Fisher’s exact test except the largest stone size, which was compared using an independent t-test. ^#^Statistically significant at p<0.05. CBD, common bile duct; CVS, critical view of safety; GB-RADS, Gall Bladder Reporting and Data System

S. no.	Parameter	Category	Total	CVS-Yes (N=35)	CVS-No (N=6)	Test value	P-value
1	Gallbladder status	Distended	28	25 (71.43)	3 (50)	-	0.632
		Contracted	13	10 (28.57)	3 (50)		
2	Pericholecystic fluid	Yes	2	0 (0)	2 (33.33)	-	0.026^#^
		No	39	35 (100)	4 (66.67)		
3	Impacted stone in neck	Yes	1	0 (0)	1 (16.67)	-	0.171
		No	40	35 (100)	5 (83.33)		
4	Largest stone size	-	-	12.50±5.77	8.95±7.78	1.32^†^	0.138
5	CBD stent in situ	Yes	3	1 (2.86)	2 (33.33)	-	0.070
		No	38	34 (97.14)	4 (66.67)		
6	GB-RADS score	2	7	3 (8.57)	4 (66.67)	-	0.010^#^
		1	34	32 (91.43)	2 (33.33)		

On analysis of various intraoperative factors, significant intraoperative predictors of failure to achieve CVS were gallbladder fundus extension up to or short of the liver edge (p=0.008), a fibrotic gallbladder bed (p < 0.005), and a positive pucker sign (p=0.025). A higher modified Nassar grade (3-4) (p=0.066), an abnormal Calot’s triangle (p=0.070), and pericholecystic fibrotic adhesions (p=0.079) were found to be borderline associated. Further details are shown in Table 8.

**Table 8:** Predictors of failure to achieve CVS: univariable analysis of intraoperative findings The data are presented as n (%). All variables were analyzed using Fisher’s exact test. ^#^Statistically significant at p<0.05. *Overdistended and contracted gallbladders were present in four patients each in the CVS-Yes group and one patient each in the CVS-No group. GB, gallbladder; CVS, critical view of safety

S. no.	Parameter	Category	Total	CVS-Yes (N=35)	CVS-No (N=6)	P-value
1	Modified Nassar grade	3-4	9	5 (14.29)	4 (66.67)	0.066
		1-2	32	30 (85.71)	2 (33.33)	
2	GB distension	Overdistended/contracted	10	8 (22.86)*	2 (33.33)*	0.120
		Normally distended	31	27 (77.14)	4 (66.67)	
3	GB fundus extension	Till liver edge	22	16 (45.71)	6 (100)	0.008^#^
		Beyond liver edge	19	19 (54.28)	0 (0)	
4	Impacted neck stone	Yes	6	5 (14.29)	1 (16.67)	1.000
		No	35	30 (85.71)	5 (83.33)	
5	Mucocele	Yes	3	3 (8.57)	0 (0)	1.000
		No	38	32 (91.43)	6 (100)	
6	Empyema	Yes	2	1 (2.86)	1 (16.67)	0.314
		No	39	34 (97.14)	5 (83.33)	
7	Adhesions					
	a) Inflammatory	Fibrotic	15	10 (28.57)	5 (83.33)	0.079
		Non-fibrotic	26	25 (71.43)	1 (16.67)	
	b) Physiological	Yes	22	17 (48.57)	5 (83.33)	0.099
		No	19	18 (51.43)	1 (16.67)	
8	Calot’s triangle	Fatty/fibrotic	11	7 (20)	4 (66.67)	0.070
		Normal	30	28 (80)	2 (33.33)	
9	GB bed	Fibrotic	10	5 (14.29)	5 (83.33)	<0.005^#^
		Normal	31	30 (85.71)	1 (16.67)	
10	Peri-cholecystic edema	Yes	10	7 (20)	3 (50)	0.332
		No	31	28 (80)	3 (50)	
11	Liver pucker sign	Yes	2	0 (0)	2 (33.33)	0.025^#^
		No	39	35 (100)	4 (66.67)	

Predictors of failure to achieve the critical view of safety

Multivariable Analysis

On multivariable logistic regression analysis, a GB-RADS score of two (adjusted OR 8.74, 95% CI 1.01-75.69; p=0.049) and a fibrotic gallbladder bed (adjusted OR 10.04, 95% CI 1.29-78.36; p=0.028) were found to be independently associated with failure to achieve CVS; the model was significant (omnibus p=0.002) with good fit (Nagelkerke R²=0.446; Hosmer-Lemeshow p=0.931).

**Figure 2:**
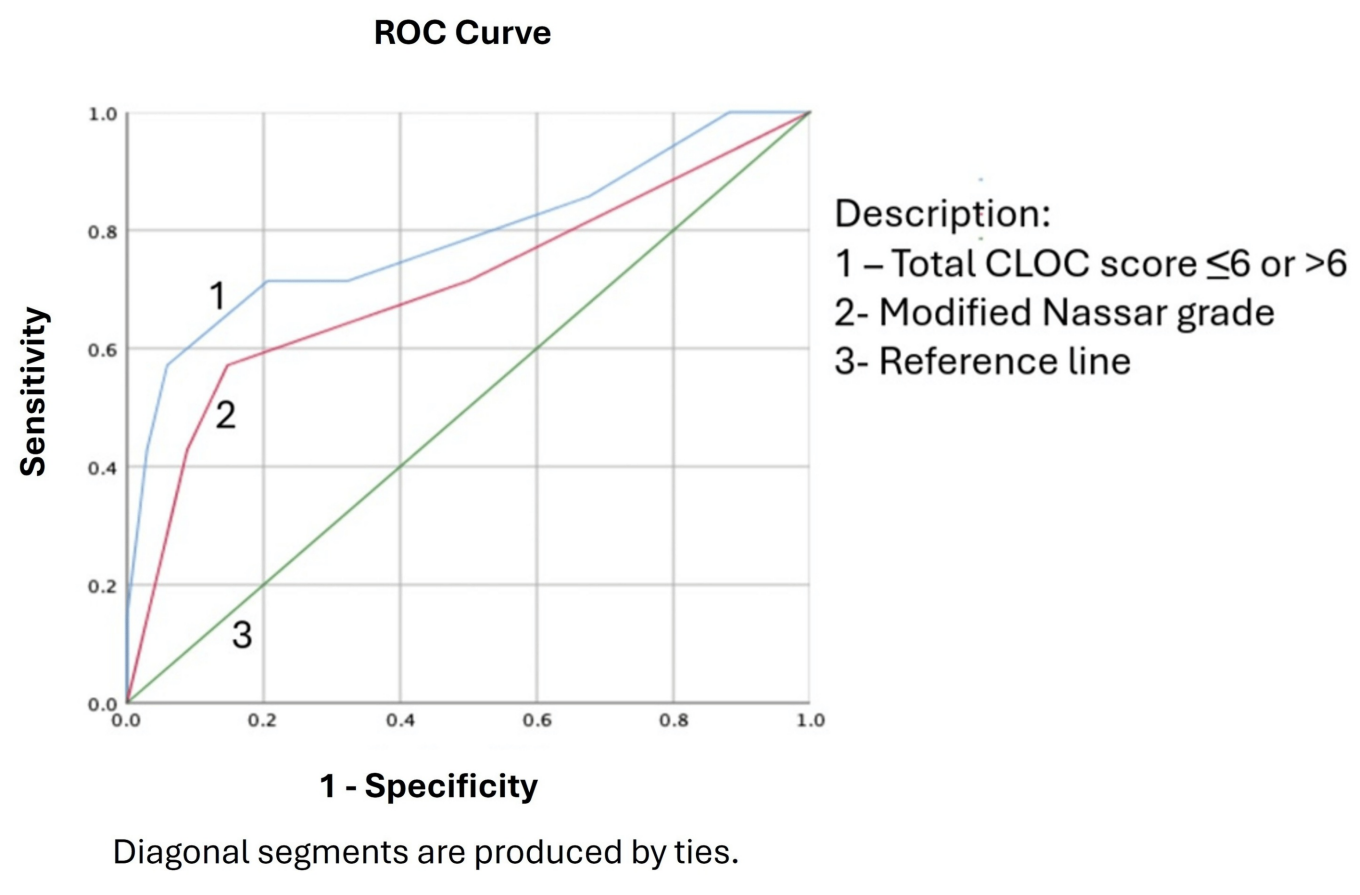
ROC curves: total CLOC score (AUC 0.788) vs. modified Nassar grade (AUC 0.704) ROC, receiver operating characteristic; AUC, area under the curve; CLOC, Cholecystectomy Laparoscopic-to-Open Conversion

In a separate score-based model, the total CLOC score was found to be independently associated with failure to achieve CVS (OR 26.6; p=0.020), whereas the modified Nassar grade was not (p=0.423). The model was significant with a good fit (Hosmer-Lemeshow p=0.745). On receiver operating characteristic analysis, the total CLOC score had better discriminative ability to predict failure to achieve CVS than the modified Nassar grade (AUC 0.788 vs. 0.704; Figure 2). A cutoff value of ≥4 for the total CLOC score was identified as optimal, providing a sensitivity of 71.4% and specificity of 79.4%. A cutoff of ≥2 for the modified Nassar grade yielded a sensitivity of 71.4% and specificity of 50%.

The relatively small number of patients in whom the CVS could not be achieved (n=6) limits the stability of the multivariable logistic regression model and increases the risk of model overfitting. Accordingly, the number of variables included in the model was deliberately restricted to minimize overfitting. Nevertheless, the wide CI suggests substantial uncertainty in effect estimation and the possibility of model overfitting. Internal validation techniques were not performed to assess model robustness because of the limited sample size and scarcity of events.

## Discussion

In this prospective study, the rate of failure to achieve the CVS was 14.63%. On multivariable analysis, a higher GB-RADS score and an abnormal (fibrotic or scarred) gallbladder bed were independently associated with failure to achieve CVS, and among structured scores, the total CLOC score discriminated inability to achieve CVS better than the modified Nassar grade. Failure clustered with markers of local inflammation and anatomical distortion rather than with demographic factors.

The CVS is a widely recommended method to ensure a safe LC. It has been shown to reduce BDI when adopted correctly [5,6]. BDI may be associated with significant morbidity, decreased long-term survival and quality of life, and increased financial burden on the patient and society, and may require multiple interventions, complex biliary reconstructive procedures, and prolonged follow-up [1-3]. Thus, it becomes imperative that the surgeon should not only strive to achieve CVS during LC, but also identify factors that may prohibit its attainment and allow early identification of difficult cholecystectomy.

Failure to achieve CVS is the earliest objective signal for a pause and adoption of a safer alternate strategy to complete the procedure [6,20]. Our 14.63% failure rate is almost identical to the 15.8% reported by Nassar et al. [11] in the largest prospective series addressing this endpoint. In a pooled analysis of over 10,000 cases, an overall CVS attainment rate of 92.5% was noted in the meta-analysis by Manatakis et al. [10]. For a standard LC, the CVS attainment rate ranged from 79.5% to 95.8% [10]. The CVS attainment rate (85.36%) in the present study lies within this reported range. The wide variation in the reported attainment rates may be due to patient selection (elective vs. emergency), operator-dependent factors (experience and attitudes), and the CVS assessment method. In the present study, all cases were electively operated on by a single surgeon, and intraoperative CVS assessment was performed in a standardized manner and in real time rather than based on review of the recorded procedure. When operative videos were reviewed objectively, all three Strasberg criteria for CVS were confirmed far less often than the operative notes claimed, with final CVS rates of only 10.8% in a study by Nijssen et al. [21] and only 15% in another study by Stefanidis et al. [22]. Against this background, a CVS non-achievement rate of 14.63% in a real-world cohort containing a meaningful proportion of hostile gallbladders is reasonable.

Many predictors of a difficult cholecystectomy have been described with an endpoint of open conversion [23,24]. Studies specifically addressing failure to achieve CVS are few [11,13,14]. Nassar et al. [11] reported higher age (over 60 years), male sex, emergency admission, past or current acute cholecystitis, previous biliary interventions, adhesions with the duodenum or colon, presence of an accessory cystic artery, gallbladder condition other than chronic cholecystitis, presence of a cholecystoenteric fistula, and higher operative difficulty (Nassar grade 3 and above) as important predictors of failure to achieve CVS. The CVS could not be achieved in nearly half (55.6%) of cases with Nassar grade 4 difficulty and in a majority (92.3%) of cases with grade 5 difficulty. A majority of cases were emergency admissions (54.9%) in this study.

Addasi et al. [13] also explored the role of surgeon-related factors in the inability to achieve CVS. In a cohort of 150 patients, the overall CVS attainment rate was 68.7%; 69.6% among consultants and 60% among residents. The rate of failure to achieve CVS was relatively high in this study. Factors associated with higher CVS attainment rates were ASA class I, emergency surgery, acute cholecystitis, and non-hepatopancreatobiliary (HPB) surgeons. Other factors such as age, male sex, diabetes mellitus, clinical frailty score, level of surgeon experience, Tokyo severity grade, and intraoperative Nassar difficulty grading scale were not significant [13]. In this study, one-third were acute cholecystitis cases, and half of these were operated on as emergencies.

In another study by Gupta et al. [14], higher age, male sex, higher ASA class, presence of Murphy’s sign, emergency surgery, neutrophil and lymphocyte percentages, gallbladder wall thickness >3 mm, and impacted gallstones on abdominal ultrasound were found to be predictors of failure to achieve CVS. However, only neutrophil and lymphocyte percentages were found to be independent predictors on multivariable analysis [14]. The rate of failure to achieve CVS was 12.8% in this cohort of 273 cases. Failure occurred due to dense adhesions, stone impaction, overhanging liver segment IVb, and anatomical aberrations (multiple cystic vessels). Emergency surgery was performed in 28.2% of patients [14].

Contrary to the above studies, there was no emergency surgery in the present study. Patients with acute cholecystitis were operated on after recovery from the acute condition. Similar to the observations reported by Addasi et al. [13], age, male sex, and greater operative difficulty as assessed by the modified Nassar grade were not associated with failure to achieve CVS in the present study.

Unlike other studies, we examined the role of the GB-RADS score in predicting failure to achieve CVS. In the present study, it was independently associated with failure to achieve CVS (adjusted OR 8.74). GB-RADS was devised as a standardized ultrasonographic language for gallbladder-wall thickening [16]; although created for malignancy risk stratification, its descriptors, including wall symmetry, layering, and pericholecystic change, are also sensitive markers of chronic inflammation. To the best of our knowledge, this is the first report to use GB-RADS to predict failure to achieve CVS. This finding is biologically coherent with earlier work identifying wall thickening and pericholecystic findings as predictors of a difficult cholecystectomy [17,23,24], with the added advantage of standardized reporting that reduces interobserver variability.

The second independent predictor of failure to achieve CVS identified in the present study was a fibrotic gallbladder bed. It was associated with nearly a 10-fold higher risk of failure to achieve CVS compared with a normal bed (adjusted OR 10.04). This is an important intraoperative finding that suggests significant inflammation precluding safe dissection in the gallbladder bed [8,9]. Diffuse fibrotic changes in the gallbladder bed or along the cystic plate prohibit safe exposure of the lower third of the cystic plate, which itself is one of the three essential components of CVS that is often neglected [4]. Thus, a fibrotic bed not only prevents the necessary cystic plate exposure but may also lead to deviant dissection cephalad toward the right portal pedicle, the route to extreme vasculobiliary injury as described by Strasberg and Gouma [25].

The three scores (modified Nassar grade, CLOC score, and GB-RADS score) were examined in this study. The total CLOC score, a six-variable preoperative composite originally derived to predict conversion [17], suggested higher odds of failure to achieve CVS (OR 26.6) and demonstrated good discrimination (AUC 0.788), performing better for this earlier endpoint than for its original target. The optimal cutoff shifted downward (four or more vs. the original >6), as expected when the endpoint moves to a more proximal event, so it is best interpreted as a risk-alert threshold. However, the large OR should be interpreted cautiously because of the limited number of outcome events and resulting statistical uncertainty. The modified Nassar grade, an intraoperative descriptor, showed only moderate discrimination (AUC 0.704) and lost independence once CLOC was included, consistent with its design as a broad measure of overall difficulty rather than a specific anatomical event [26]. GB-RADS adds a standardized preoperative imaging signal available at the time of ultrasound reporting. Taken together, these findings suggest that a pragmatic two-stage approach of preoperative risk stratification with the CLOC and GB-RADS scores, followed by intraoperative use of the modified Nassar grade, may be explored further to validate its clinical utility.

Several additional findings were associated with failure to achieve CVS on univariable analysis, although they were not evaluated as independent predictors because of event scarcity and should therefore be regarded as red flags deserving further evaluation. These included the presence of pericholecystic fluid, the pucker sign, limited gallbladder extension (i.e., the fundus not extending beyond the liver edge), fibrotic adhesions, an abnormal Calot’s triangle, prior ERCP, and modestly elevated cholestatic enzymes (alkaline phosphatase (ALP) and GGT), all of which may be considered surrogate markers of inflammation [23,27,28].

The liver pucker sign is an important intraoperative finding. It suggests chronic fibrosing inflammation leading to shortening of the cystic plate with subsequent difficulty in gallbladder dissection along its bed and an inherently higher risk of right pedicle injury [19,25,27]. This anatomical alteration may also explain the observation of the gallbladder fundus being confined within the liver edge. This finding also hampers proper gallbladder fundus retraction and may therefore lead to difficulty in achieving CVS satisfactorily.

Several known predictors of difficult cholecystectomy, such as older age, male sex, higher BMI, acute cholecystitis at presentation, elevated inflammatory markers, and impacted gallbladder stones, were not found to be significant in the present study. This most likely reflects the composition of our cohort (predominantly young and female, a narrow BMI range, and a policy of interval rather than emergency cholecystectomy) together with the small event count, rather than any contradiction of the published evidence [11,24].

Clinical implications

Preoperative assessment using GB-RADS and CLOC scores may help identify patients at higher risk of failure to achieve the CVS during LC and may assist in appropriate preoperative planning and risk stratification. However, the clinical effectiveness of management modifications based on these scores has yet to be established and requires further evaluation in larger prospective studies.

Intraoperatively, a scarred gallbladder bed, gallbladder fundus confined within the liver edge, presence of the pucker sign, dense pericholecystic adhesions, or a higher Nassar grade should trigger a time-out, deliberate reassessment, optimized retraction, a second opinion, and liberal use of imaging [6,12,29], and, if CVS remains unattainable, an early bailout, rather than persistent dissection in a hostile plane, should be strongly considered [6,12]. Inability to achieve CVS is not a failure of judgment; rather, failure to recognize that it cannot be achieved is. That all six failures were completed safely by STC with no BDI suggests that STC serves its purpose as a bailout procedure in preventing BDI [30].

Strengths and limitations

Strengths include a clinically meaningful endpoint tied directly to BDI prevention, prospective multi-domain data collection, a single specialist hepatobiliary surgeon ensuring a consistent standard for declaring CVS attainment, and the use of three structured scores enabling direct comparison with the literature. Unlike other studies, we specifically examined the predictive role of perioperative parameters such as GB-RADS, the relationship of the gallbladder fundus to the liver edge, presence of the liver pucker sign, gallbladder bed status, and the nature of Calot’s triangle, which act as surrogate markers of inflammation and have not been specifically examined in previous studies.

While the performance of all procedures by a single surgeon in this study reduced technical heterogeneity, it was also prone to interobserver variability with respect to the assessment of the CVS and other important intraoperative findings. To overcome this, the CVS was considered achieved based on an objective scoring system, and only after a time-out session and discussion with another surgeon (a senior fellow) of the operating team, who was well aware of the concepts of safe cholecystectomy. Other relevant intraoperative findings were also confirmed in a similar fashion. The approach adopted also allowed independent review while minimizing interobserver variability.

The principal limitations are the nonrandomized study design and the small sample size (n=41) with only six failure events, which restrict the number of variables that can be modeled and produce wide CIs; sparse categories, single-center conduct, a female-predominant cohort, and an elective/interval-surgery policy with exclusion of acute cases further limit power and external generalizability, and the most important complication, BDI, could not be studied. These findings should therefore be regarded as clinically credible and hypothesis-generating, requiring confirmation in a larger multicenter cohort that includes emergency cholecystectomy.

## Conclusions

The CVS can be achieved in a majority of LCs. The inability to achieve the CVS may be associated with inferior perioperative outcomes compared to when the CVS is achieved. A higher GB-RADS score, a fibrotic gallbladder bed, and a higher total CLOC score suggest a higher risk of inability to achieve the CVS. The presence of pericholecystic fluid, a liver pucker sign, and a gallbladder fundus confined within the liver edge are additional factors that may suggest a higher risk of not being able to achieve the CVS. These radiological, intraoperative, and composite-score findings may aid in identifying patients at increased risk of failure to achieve the CVS during LC and, thus, may facilitate preoperative risk stratification and operative planning. Although the identified predictors demonstrated significant associations with failure to achieve the CVS in this cohort, these findings should be considered exploratory in nature, given the study limitations. External validation in larger prospective studies is warranted before these predictors can be incorporated into routine clinical decision-making.
